# Supplementary material for: Patterns of Intron Gain and Loss in Fungi
Source: PLoS Biol. 2004 Nov 30;2(12):e422. doi: 10.1371/journal.pbio.0020422 (PMC532390; doi:10.1371/journal.pbio.0020422)
Supplement: Table S1 — Also available at http://genes.mit.edu/NielsenEtAl/. (4.3 MB ZIP). [file pbio.0020422.st001.zip › NielsenEtAl/html/1339.html]

AN1484.1.NCU01965.1.MG04396.1.FG06850.1


```
 CLUSTAL W (1.82) Multiple Sequence Alignments - Introns Inserted


Sequence 1: MG04396.1	1192 aa
Sequence 2: FG06850.1	1093 aa
Sequence 3: NCU01965.1	1093 aa
Sequence 4: AN1484.1	1294 aa
Alignment Length: 1514 aa
Number Identitical Residues: 415 aa
Alignment Score (without introns) 23844


MG04396.1 	MDKRSRLLLAPKVDAHLRDAIVCADGYARRPIERSDISL0ITSSPARPPAIGLHQDPPKN
NCU01965.1	---------------------------------------~--------------------
FG06850.1 	---------------------------------------~--------------------
AN1484.1  	---------------------------------------~--------------------
          	                                                            

MG04396.1 	RLGSTQSTKGAQASPAGVAPAMGCGGAVWLWKHSIFKLC2GRGNPI1GSTKGPDDGTPPP
NCU01965.1	-------------------------MPLSLWRLAIGS-S~RRHSLL~KITSTRVLPVPRP
FG06850.1 	----------------------------------MATNS~GRGNPI1GASEGIQDATSTP
AN1484.1  	----------------------------------MAQDS~ASMNPT1GEPTGPKVDVPPV
          	                                  :   .    .     .      ..  

MG04396.1 	LSQDTKSAIQETTSTGNPGTHAAGQDAGAAEA---PAKVKTAKEL1EKERKKAEKQAKFE
NCU01965.1	YSTYSNNKKVIMPGE----DQPAAAPAAAPAAQDAPGAPAPKKND1KKEKAKADKAAKFA
FG06850.1 	PAVPAQEKKEIAQST--TETHASGQDAGAAAATDGPPKVKTEKEL1EKERKKAEKQAKFE
AN1484.1  	GNNGQQN---------------AGQDG-------AAPKVKTEKEL1ERERKKAEKLKKFQ
          	     :.               :.  .       ..    . *:  ::*: **:*  ** 

MG04396.1 	QKKAAAASKAAPAAGADKPKKEKAKKAEEPVLPPYENDTPPGEKKRLRPLTDPHYTAYNP
NCU01965.1	AKQAAAKAKQPAAQ-----SAPKEKKEKTPALPPYEDSTPAGEKKVIQSFEHPHFSAYNP
FG06850.1 	AKKQKAAVAAPKAP---KEKKPKEKKAEEEPIPEYVEDTPKGEKKRIRSFDDPHFKAYDP
AN1484.1  	EKQAKAAAKT---------------------------TTPKAEKKAPKVEKDKTADAYDP
          	 *:  *                                ** .***  :   .    **:*

MG04396.1 	VAVESAWYDWWEKEGFFKPEFTKDGKVKDEGSFVIVHPPPNV1TGALHMGHALGDSLQDL
NCU01965.1	SAVEAAWYQWWEKAGYFKPESCRK---PSAGKFVIPLPPPNV~TGALHCGHALANSLQDT
FG06850.1 	IAVESAWYDWWEKEGFFKPEFKPDGNIKDEGKFVIVHPPPNV~TGALHMGHALGESLQDL
AN1484.1  	KVIEAGRYQWWEERGLFKPEFGPDGKVKPEGYFVIPIPPPNV~TGSLHMGHALTNALQDT
          	 .:*:. *:***: * ****   ...    * ***  ***** **:** **** ::*** 

MG04396.1 	MIRYERMRGKTTLWVPGCDHAGIATQTVVEKMLWKSEQKTRHDLGRPDFISKVWEWKGEY
NCU01965.1	LIRWYRMKGYETLWVPGCDHAGISTQSVVEKMLWKKEKKIRQELGREKFTDLVWEWKGEY
FG06850.1 	MIRWSRMQGKTTLWVPGCDHAGISTQSVVENMLWRRHGLTRRDLGREKFVNTVWEWKEDY
AN1484.1  	MIRWQRMKGKTTLWLPGMDHAGISTQSVVEKMLWKKEKKTRHDLGRKAFLERVWDWKHEY
          	:**: **:*  ***:** *****:**:***:***: .   *::***  * . **:** :*

MG04396.1 	HEKINNALRKMGGSFDWSREA~FTMDENSTAAVMEAFVRLHEEGIIYRANRLVNWCTQLN
NCU01965.1	HQRINNAQKLMGGSMDWSREA~FTMDKNLTAATMETFCRLHDEGLIYRSNRLVNWCTHLN
FG06850.1 	HKRINNTLCKMGGSYDWSREA1FTMDKNLSAAVTETWVRLYEEGTIYRANRLVNWCTKLN
AN1484.1  	HGNIGNALRRVGGSFDWTREA~FTMDDNLSAAVTETFVRLHEEGIIYRANRLVNWCVALN
          	* .*.*:   :*** **:*** ****.* :**. *:: **::** ***:*******. **

MG04396.1 	TAISNLEVENKEIAGRTLLDVPGYAKKVEFGVIVYFKYPLEGSDETITVATTRIETMLGD
NCU01965.1	TALSGLEVETKEITGRTLLDVPGYDKKVEFGVLTHFKYQIDGSEETIEVATTRPETMLGD
FG06850.1 	TTLSNLEVVNKELTGRTLLDVPGYDKKVEFGVIVHFKYPVEGSDELIEVATTRIETMLGD
AN1484.1  	TSLSNLEVENKEVEGRTLLDVPGYEKKVEFGVLTHFCYEIDGTKERIEIATTRPETMIGD
          	*::*.*** .**: ********** *******:.:* * ::*:.* * :**** ***:**

MG04396.1 	TG1IAVHPDDERYKHVIGKN~AVHPFIPGRKLPIVADKSVEMDFGTGAVKLTPAHDPNDF
NCU01965.1	TG~IAVNPEDPRYTHLVGKF~ARHPFVD-RLLPIVTDNYVDKEFGTGAVKLTPAHDFNDY
FG06850.1 	TG~IAVHPKDDRYKHLIGKT~AVHPFIEGRKLPIVADEYVDREFGTGAVKLTPAHDPNDF
AN1484.1  	T-~----------------A1ARHPFID-RLLPIVADPEVDPEFGTGAVKITPAHDFNDF
          	*                    * ***:  * ****:*  *: :*******:***** **:

MG04396.1 	AMGQTHKLEFINILTDDGLMNENAGAYKGQKRFDVRYTIQDDLKKAGLYVDKKDNPMSLP
NCU01965.1	QLGQRHNLEFINILNENGTLNDNAGPFKGQKRFDARYTVVEELTKLGLFVKKEPNPMKIP
FG06850.1 	TLGQKHGLEFINILTDDGLMNENAGAYKGQKRFDVRYTIQDDLKAKGLYVDKKDNAMKVP
AN1484.1  	NRGKAHNLEFISVLNDDGTFNKNGGPFAGMKRFDARYKVIEMLKEKGLYVKWEHNPMKIP
          	  *: * ****.:*.::* :*.*.*.: * ****.**.: : *.  **:*. : *.*.:*

MG04396.1 	MCERSKDIIEPIMKPQWWMRMREMADAAIGAVEDGSIKIRPQSSEKSFKHWMRNIQDWCI
NCU01965.1	LCEKSKDVIEPMMTEQWWVRMKEMGEAALQVVEEGKVKISPESATKSYKRWLADIQDWCI
FG06850.1 	LCDKSKDIIEPIMKPQWWVRMKELAEPALAAVRNGSIKIRPESAEKSYYRWLENIQDWCI
AN1484.1  	RCAKSNDVIEPILKPQWWMKMESLAEPAIKAVENGEIIIRPESAEKSYFRWLRGINDWCL
          	 * :*:*:***::. ***::*..:.:.*: .*.:*.: * *:*: **: :*: .*:***:

MG04396.1 	SRQLWWGHQCPVYYAEIEGDENDRGANN-RWFAGKTEQEAEEKAKKALGEKKFKLVRDDD
NCU01965.1	SRQLWWGHRIPAYRVIFEGEEGQRENEKSEWVVGRTQEEAQAKAEAKFAGRKFTLEQDPD
FG06850.1 	SRQLWWGHRSPVYFAKIEGGAEDIPEEK-LWFAGRTRQEAEEKAKAALPGKTYTLEQDED
AN1484.1  	SRQLWWGHQAPAYFVQIEGEEGDDSDGN-LWVTGRNEEEAQKKAEAKFPGKKFTLKRDED
          	********: *.* . :**   :    :  *..*:..:**: **:  :  :.:.* :* *

MG04396.1 	VLDTWFSSGLWPFSTLGWPNTSAEDLQKLYPTSTLETGWDILTFWIARMIMLGMKLTGKV
NCU01965.1	CLDTWFSSGLWPMAILGWPNTENLDFKKFFPTSMLETGWDILFFWVSRMIMLSLKMTGEV
FG06850.1 	VLDTWFSSGLWPFSTLGWPN-QTHDLEKLYSTEVLETGWDILFFWIARMIFFGIKLTGKI
AN1484.1  	VLDTWFSSGLWPFSTLGWPR-QTHDFENLYPTSVLETGWDILFFWVARMIMLGIKMTGKV
          	 ***********:: ****. .  *:::::.*. ******** **::***::.:*:**::

MG04396.1 	PFNEVYCHSLVRDSDGRKMSKSLGNVIDPLDVIKGINLDDLHAKLLVGNLASTEVEKATK
NCU01965.1	PFTEVYCHSLIRDSEGRKMSKSLGNVIDPLDIIRGIELEDLHAKLLVGNLKEEEVARATK
FG06850.1 	PFKEVYCHSLVRDSEGRKMSKSLGNVIDPLDIIAGVELQTLHDKLTQGNLDPKEVAKATK
AN1484.1  	PFKEVYCHSLIRDSEGRKMSKSLGNVIDPLDVMEGIKLEDLHAKLLVGNLAEKEVATATK
          	**.*******:***:****************:: *::*: ** **  ***   **  ***

MG04396.1 	YQKQAFPQGIPECGADALRFCMINYTTSG~GDINFDIKVMHAFR~RFSNKIWQASKYVLG
NCU01965.1	YQKTAFPGGIPECGADAMRFTLLSYTTGG~GDINFDIRVMHAYR~RFCNKIWQASKYVLG
FG06850.1 	YQKTAFPDGVPQCGADAMRFTMINATTGG~GDINLDVKIIHGYR~KFCNKIFQATKYVLG
AN1484.1  	YQKKAFPKGIPECGADALRFSLVSYTTGG1GDIAFDIQVIHGYP1---------------
          	*** *** *:*:*****:** ::. **.* *** :*::::*.:                 

MG04396.1 	KLENYPDFVPREKRTLGGSESLAEQWILHKMNQATKGINEALQEREFMKSSQLVYAYWYN
NCU01965.1	KLP--QDFMPKGELDTAN-FSVPEKWILHRMNVAVKGMNEALEAREFSRATKVAYQFFYD
FG06850.1 	SLP--QDFTP-SKSGIARGETLAERWILHKMNSAAKEINRALEDREFSKSTLIVYRYWYN
AN1484.1  	-----------------------------------------LEDREFSHAASTVYQYWYS
          	                                         *: *** :::  .* ::*.

MG04396.1 	QLCDVFIENSKALLQDGSPAE-QESAMQTLYTALESALVLIHPFMPFVTEELWQRLPRRK
NCU01965.1	ELCDVFIENSKGILSDGTPEE-QQSVQQTLYHALDVALRLLHPIMPYITEELWQRLPRKQ
FG06850.1 	ELCDVYIENSKAIIRDGTEKE-RESAIQTLYTALETALTMIHPFMPFITEEMWQRMPRRP
AN1484.1  	QLCDVFIENSKSLLAPELPQEVQESAKQTLYTALEGALTMIHPIMPFITEELWQRLPRRP
          	:****:*****.::      * ::*. **** **: ** ::**:**::***:***:**: 

MG04396.1 	GDTTPSLVVASYPQYDEALDNASAEIAYELVLDCSKGIRSLMAEYALKDEAQV1FIQCYD
NCU01965.1	GD-GETIMLAPYPAFESQLEFATEAEDYELGLKCAGGIRSLAADYNIKSDGRA~FIKATT
FG06850.1 	EDQTKSIVVAKYPTYNEKLDDPESERAYELVLGCSKAARSLMAEYALKDEAEV1IIQAYN
AN1484.1  	GDNTISIMKAKFPEYKAEFDDSTAETAYELILNTSKAIRSILAQYDVKTKGDV~IVQTYD
          	 *   ::: * :* :.  :: .     *** *  : . **: *:* :* .. . :::   

MG04396.1 	DASVATARKELQSIKSLSGKGVTAVQVLEASAKRP-AGCVVFPCGSTAAVFLHVKGRV-D
NCU01965.1	ADSLATVSAQLAAIRTLCGKGVKEVNVLGADEELP-RGCAVYVINAEITVLLQVGGSISD
FG06850.1 	DTSLATVKEQASSIKSLSGKGIKGVEILGSDASRP-AGCVAYPVSTEASVFLHVKGRV-D
AN1484.1  	ATSHKTVSDELHIVKSLGGKTLGELSVLGPENTTPPSGCVVAPVGSQAAVYLRVSKEVAL
          	  *  *.  :   :::* ** :  :.:* ..   *. **..   .:  :* *:*   :: 

MG04396.1 	IDAEIAKAQKKLDRASGQIDRQKKVLNDPKYQEKVTAE~LQATEK-----~---------
NCU01965.1	IDAEIKKITTKLQKTDLTIKKQQELLSKDG-FEKVSEA~VQESEK-----~---------
FG06850.1 	IDAEIIKAQKKLDKAKSSIQKQEKILNDPGYLEKVSDA~IRETDE-----~---------
AN1484.1  	EQEEKGKAS--LKKAQETVRRQTTLINSAGWKEKAKPE0HVLSDKNASRS0AFVNGKSLH
          	 : *  *    *.::.  : :*  ::..    **..      :::.:: : :  ...:  

MG04396.1 	-----KRLADMESEAEG--------------------FKATIKQFEDLKLE---------
NCU01965.1	-----QKLADAQAAKEN--------------------YQRTLEEFSKLKI----------
FG06850.1 	-----KRLSDAKQEMNS--------------------FEETIKQFEQLKLE---------
AN1484.1  	RKPKNPRTARILKAKEPQLIEGAKRVLLLHGSKCPTPLHTVLKVFHSLTTPHSVLFHKKN
          	 .... : :      : .  ..:.      .:...:. . .:: * .*.   :    ...

MG04396.1 	------------------------------------------------------------
NCU01965.1	------------------------------------------------------------
FG06850.1 	------------------------------------------------------------
AN1484.1  	ENIHPFESTESLEFLANKNDCGMVIFGSSNKKRPNCLTIARIFDSKVLDMAELLLLPDAN
          	..  . .::.: .  :......    .::... ... : :   .:.  . :.    ..:.

MG04396.1 	------------------------------------------------------------
NCU01965.1	------------------------------------------------------------
FG06850.1 	------------------------------------------------------------
AN1484.1  	GEGIPEMNRLSMHVAIGLRPLMLFSGSAWDDTTSTTHTMLKSMLVDLFKGETSDKIDVEG
          	... .. .  :   : .  .    :.:: ..::::: :  .:   .  ...::.. . ..

MG04396.1 	------------------------------------------------------------
NCU01965.1	------------------------------------------------------------
FG06850.1 	------------------------------------------------------------
AN1484.1  	LQYALMVGAEEPTAGLAPIIHLRWYKIVTKRSGHKLPRVELEEIGPKLDFKVGRIQEAPR
          	 . :   .:...::. :.       .  :. :. . .  . .. ... . . .  ..:. 

MG04396.1 	------------------------------------------------------------
NCU01965.1	------------------------------------------------------------
FG06850.1 	------------------------------------------------------------
AN1484.1  	DVMKEAMKQGKKPNEEIKNKKNIGIDLIGDKVGRVHLAKQDLGGLQTRKMKGLKRRAGVE
          	.  ..: ......... ..... . .  ... .    :... .. .: . .. .  :. .

MG04396.1 	---------------------------
NCU01965.1	---------------------------
FG06850.1 	---------------------------
AN1484.1  	SDDEGDADMMDVDEVSEDEKTKRARHD
          	:.....:.  . .. :....:. :  .
```
